# Knowledge in identifying venomous snakes and first aid methods of snakebites among nursing students: A cross-sectional study

**DOI:** 10.1371/journal.pone.0299814

**Published:** 2024-04-04

**Authors:** Isuru Jayathilaka, Eranthi Weeratunga

**Affiliations:** Faculty of Allied Health Sciences, Department of Nursing, University of Ruhuna, Matara, Sri Lanka; BOKU: Universitat fur Bodenkultur Wien, AUSTRIA

## Abstract

**Background:**

Snakebites are a dangerous and significant medical emergency that occurs worldwide. The World Health Organization has recommended that teaching and training in the prevention and management of snakebites be included in the curriculum of nursing schools and other educational activities. Identification of venomous snakes and first aid would be more critical in the prevention of occupational danger worldwide. This study aims to assess the knowledge in identifying venomous snakes, snakebites, and first aid methods of snakebites among nursing students in the Southern Province of Sri Lanka.

**Methods:**

A descriptive, cross-sectional study was performed among 425 nursing students who were studying in different educational settings: undergraduates at the University of Ruhuna, and nursing students in the three schools of nursing in Galle, Matara, and Hambantota. Data were gathered by incorporating a pre-tested self-administered questionnaire after obtaining institutional permission. The total score of whole knowledge ranged from 0 to 34 for the identification of venomous snakes. Data collection was performed after obtaining ethical clearance from the Ethics Review Committee, Faculty of Allied Health Sciences, University of Ruhuna, Sri Lanka.

**Results:**

Most of the students (82.6%) were in the 24–26 age category and the majority were females. Most of the sample (64.7%) had low knowledge of identifying venomous snakes. A higher percentage of students (57.4%) had a sufficient level of knowledge about first aid methods associated with snakebites and 169 participants (39.8%) had a high level of knowledge regarding first aid methods. Further, a significant impact on students’ knowledge and knowledge of first aid methods was reported.

**Conclusion and recommendation:**

The overall knowledge of identifying venomous snakes among the nursing students was inadequate. However, the knowledge about the first aid methods was at a moderate level. Strategies are needed to improve knowledge in identifying venomous snakes and first aid methods of snakebites amongst nursing students in both educational settings.

## Introduction

Snakes are considered reptiles and are believed to be “cold-blooded” animals, which plan to retain body temperatures around that of their environment and essential warm weather to become active hunters. Also, snakes pursue shelter in zones where the temperatures are preserved beyond freezing. Appropriate shelters for those snakes could be various spaces like below ground, under rocks, in holes, or any buildings made by humans that are used as temporary accommodation [1].

The bite of a snake, mainly a venomous snake is defined as a snakebite [2,3]; it can be either venomous or non-venomous mostly. These venomous snakebites should be a concern as a medical emergency [3]. Bites from venomous snakes rapidly show different features such as signs of inflammation and staining of the adjoining tissue; paralysis that may stop breathing, bleeding causing a fatal hemorrhage; and permanent kidney failure, and tissue damage which lead to everlasting disability and amputations of limbs [4]. Other symptoms are nausea, tingling feeling, lack of muscle activities, increased pulse, and activity failures/weakness. Non-venomous snakebites may not be dangerous; the one factor may be potential infection/illness [1,4].

Among the neglected tropical diseases (NTD), snakebite envenoming is significantly underreported worldwide each year and reported permanently disabled victims [5]. The annual number of snakebites worldwide is expected to be between 1.2 and 5.5 million [3,6]. In many tropical and subtropical countries, it is an important and neglected public health problem such as in tropical regions of South Asia, South-East Asia, Sub-Saharan Africa, and Latin America [7]. Further, Kasturiratne et al. [7] reported that snakebites trigger considerable morbidity and mortality worldwide; with the highest burden occurring in South Asia, Southeast Asia, and sub-Saharan Africa [3]. Also, snake bites are largely common in rural people in settings with a lack of resources, and mainly among low-cost and/or non-mechanical farming areas and other sectors/jobs such as among workers in agriculture, women, and children. Also, largely common in rural people, settings with a lack of resources, and mainly among low-cost and/or non-mechanical farming areas and other sectors/jobs such as among workers in agriculture, women, and children are most bitten by snakes [3]. In many countries, snakebites are a usual medical emergency and a cause for hospitalization. In addition, venomous snakebites cause many amputations and other major health issues such as infections and mental conditions as mentioned earlier [3].

However, due to a lack of adequate reporting in practically every corner of the world, the full range of the problem is unknown [4,8]. The fact sheet on snakebites by the World Health Organization (WHO) reported that more than 50,000 persons die each year due to snakebites, an average of three times larger than amputations and other enduring disabilities found annually [4]. Venomous snakes residing close to urban and rural regions can be extremely dangerous if they meet people, pets, or livestock [1]. Due to its large population density, broad agriculture activities, and the presence of numerous venomous snakes, countries are differently affected. The Southeast Asia Region is one of the most greatly affected regions in the world [4]. The poisonous snake creatures of South-East Asia are varied and abundant. Hence, tens of thousands of healthy young people, particularly those engaged in farming and plantation work, die or become permanently disabled due to snakebites [4]. In addition, snakebites are highly increased during floods [5] and India has evidence for such increased snakebites [5]. In America, reptile envenomation has increased after the hurricanes and post-storms [9].

In Sri Lanka, an average of 37,000 snakebites are reported annually [7]. There are numerous snake species in Sri Lanka. Over 40,000 cases of accidents involving snakebites occur each year in various agroecological regions [10]. Snakebite is mostly an occupational danger in Sri Lanka, affecting people in rural areas [3,11,12], especially associated with farming [6,13], as it is in other countries. The highly venomous Cobra (*Naja naja*), Russell’s viper (*Daboia russelii*), and Kraits (*Bungarus ceylonicus*) are accountable for much of the morbidity and mortality (95%) of snakebites in Sri Lanka [11]; while the King cobra, Indian kraits, and Russell’s viper were the highly venomous snakes reported in India, [13] in Sri Lanka, the Hump-nosed viper (*Hypnale zara*) is responsible for about 27% of all snakebites [3,11,14,15].

The medical and healthcare workers’ training should include identifying medically necessary snake species, clinical diagnosis, and the correct usage of antivenoms and other treatments [3]. It is recommended that snakebite prevention and management be included in medical and nursing curricula and addressed clearly by creating special training courses and other educational activities based on national standards and guidelines [4,16].

The exact identification of snakes and snakebites is very important to employees in healthcare to identify and treat casualties with snakebite poisoning [17] due to the biomedical significance of reptiles. Further, snake recognition is crucial for the public, mainly those who reside in areas with a high prevalence of snakebites. Due to the significant variety of snakes and the insignificant changes among some species of snakes, the proper detection of these snakes is the most complicated.

Geographic distribution of seriously venomous snakes can be a useful aid to identification. Such lack of identification may have been accountable for several mortalities although there is has antivenom treatment [18]. For best treatment and management, the identification of the snake species that caused to bite is crucial. This can be done by the expert identification or interpretation from the subsequent "clinical syndrome" of a dead snake or an image (photographs taken from mobile phones, etc.) of a snake. Syndromic techniques and algorithms to diagnose the snakebite species in various areas of the region are recommended [4]. Deep Learning Model (DLM) for identifying snakes by using snakebites is reported by Kamalraj [13]. Also in India, snake attacks and treatment for snake poisons were a very big issue. Similarly in Sri Lanka, 103 snake species are found and difficult to differentiate. Therefore, this DLM was used to differentiate such types of issues of snakes [19].

The detection and identification differences of snake venom are greatly required for clinical treatment on time [20]. Snake venom causes serious disabilities, and it is one of the reasons for high incidences and deaths worldwide [20]. A survey done at the National University of An-Najah, Palestine revealed the knowledge of the identification of snakes by 200 nursing students; 75.5% of students correctly identified *Vipera palaestinaeas*, the most common venomous snake in Palestine [21]. Another research team in Sri Lanka proposed to use the DLM as an approach to differentiate various types of snakes (to identify snakes using images) [19]. These deep neural networks have grown into a popular technique for image recognition. In Sri Lanka, there are 103 snake species discovered. Many have common features such as body-head shape, color patterns, and other physical attributes which make it hard to recognize most snakes individually. Further, they mentioned that the Saw Scaled Viper, Common Krait, Russell’s Viper, and Indian Cobra were the four highly venomous snake species, and the Hump Nosed Viper was taken as the moderately venomous snake in Sri Lanka.

Hence, the detection of snakes is a big issue for employees in healthcare and the local people, etc. [17]. To solve this issue, this research team has developed a special system in Sri Lanka called the web-based snake identification service (e.g., Sri Lanka- Snakebite identification) which delivers a very quick and perfect recognition by experts (e.g., herpetologists). Most appeals were from the main city, Colombo District/Sri Lanka (28.8%) [140], and 63 (13.0%) of snakes were classified as medically vital snakes. Most of the inquiries (80.0%) [389] were linked to either too weakly venomous or non-venomous species. Most of the requests were the inability to recognize the snakes (283, 58.2%). For those who tried to detect snakes, only 59 (12.1%) of the sample were able to do the correct identification, whereas 144 (29.6%) of the sample had recognized the snake incorrectly. Hence, this web-based snake detection service offers an example of a productive and helpful type of quick detection of snakes. Parallel guides could be employed in other countries to provide exact identification of snakes among healthcare workers and the public [17].

When considering first aid methods, many venomous snakebites should be considered life-threatening. When somebody has been attacked by a venomous snake, time is an important factor [1]. Both Western and traditional methods are the most common first aid methods for snakebite treatment and result in extra risk than benefit. Though traditional healers and practices are appreciated in many communities, timely delivery of envenomed people to the hospital is very important. Reassurance, using a pressure pad over the bite wound, immobilization of the bitten limb, and transportation of the victim to a location where they can get immediate medical attention are all recommended first aid treatments [4].

Envenoming and fatalities/deaths leading to snakebites are mainly valuable public and health problems that occur in rural tropical areas. Residents in these areas encounter high prevalence and deaths because of inadequate approaches to healthcare services and a shortage of antivenom. Many victims stay alive with permanent physical problems due to some tissue necrosis and mental issues to a certain extent. Since most snakebite persons are young, their disabilities could impact their income status and indirectly affect the country’s economy. Hence, snakebite has not obtained the attention of the national and international healthcare authorities. Due to this reason, it is considered a neglected tropical disease [7].

Envenomation due to snakebite is a miscalculated and ignored/neglected public health problem responsible for considerable disease and death and socio-economic issues of disadvantaged people in rural and tropics such as Asia, Africa, Oceania, and Latin America. In developed countries, snakebite mostly occurs due to the recreational activities of people, while in developing countries it is considered an occupational injury and highly affects young persons engaged in agricultural work, largely men. Poor healthcare services, delays, lack of administration of antivenom, and obstacles with transportation between rural settings and healthcare centers are major contributing factors and the cause of the high number of deaths [22].

A cross-sectional survey was done to evaluate the level of knowledge on first aid methods among nursing students in Palestine towards snakebites using 200 nursing students at An-Najah National University. According to the results, 29% of students thought their snakebite knowledge was good. Only 42% of nursing students obtained certain training about snakebites. Findings revealed that with knowledge of first aid, many of the students identified that requesting the affected person to stay quiet is important (85%) and that victims should be sent to the hospital/healthcare setting as early as the snakebite (74.5%), and only 15.5% recognized that administering an ice/cool pack to the bite site was not working [21]. Another study was conducted in Savannakhet Province-Lao, among 119 healthcare providers to assess knowledge about snakebite management [23]. According to their research study, 52.1% were physicians, and 47.9 were nurses. Healthcare workers (52.1%) had less than ten years of experience, 15.1% had 10 to 20 years, and 32.8% had more than twenty years. Most healthcare workers (66.4%) said they had never received snakebite management training. Findings were that knowledge of first aid such as fixing incisions, doing suction, and giving massage to the bite site, compression or tourniquets, and intra-muscular analgesics were not indicated as treatments for patients with snakebites, according to most study participants. Following snakebites, 88.2% correctly answered that immobilizing the person and the damaged limb and transporting them to the closest hospital as early as possible are the proper management processes [23].

Another survey was done to measure the knowledge of the management of snakebites in healthcare service suppliers in Bangladesh rural communities [24]. It was found that around 72.0% to 94.0% of doctors could reveal the knowledge of features of venomous snakebites; nurses around 50% to 90%; and other practitioners 54.0% to 89.0%. According to the statistics, the season with heavy rain was the most common time for snake bites in Bangladesh’s rural regions. A lack of knowledge, skill, and experience in treating snakebite victims were the reported final consequences of this study [24].

Snakebites account for the high disease burden in developing countries and it is responsible for underrated medical and surgical emergencies. Due to the limitation of resources in those countries, management of snake envenomation was a public health challenge [16]. Therefore, it is a critical need to obtain knowledge envenomation of snakebites around the world and to encourage public health guidelines directed at developing the management and prevention of snakebite envenomation. The geographical arrangement is also very important to identify highly prevalent areas of snakebites. Thus, these areas require more awareness sessions and developed training of medical staff on first aid and other needy care for snakebite patients, to contribute to the decrease of morbidities and envenomation [25]. More snakebite training is needed in the province owing to the absence of awareness of the treatment of snakebites among healthcare providers. To guarantee that medical professionals have the knowledge and abilities to treat snakebites, universities, and colleges of health science should plan snakebite management/treatment in their program curriculum [23]. Not only medical personnel, but nursing students also must work with patients with snakebites. The awareness about how to deal correctly and scientifically with snakebite situations and the identification of snakes/snakebites would be more important for nursing students [21].

Limited but reliable data are accessible from the rural-tropical areas where snakebites appear in most instances. Therefore, envenoming characteristics in different regions, the true global incidence of snakebite envenoming, and its impact were largely unidentified. No research study was done in Sri Lanka regarding the knowledge of the identification of snakes/snakebites and first aid methods of snakebites among nursing students. Hence, it is worth having such findings as nursing students have to work with patients with snakebites in different clinical settings. When their knowledge about identification and first aid methods is improved, future identification and snakebite management will also be improved. Further, these findings would help to minimize the lack of knowledge among nursing students, and it will be important to include the identification of venomous snakes in the current curriculum and revise the curriculum on first aid methods of snakebites as suggested by the WHO. Therefore, it is very important to assess knowledge of the identification of venomous snakes/species, and first aid of snakebites among nursing students in Sri Lanka. This study will be useful in the future for their educational purposes once added to the curricula and practices in the clinical setting. The main aims of this study were to assess the knowledge in identifying venomous snakes and first aid methods of snakebites in nursing students in the Southern Province of Sri Lanka.

## Materials and methods

### Study design

A cross-sectional, descriptive design was used to assess the knowledge in identifying venomous snakes and first aid methods for snakebites among nursing students in the Southern Province of Sri Lanka.

### Study setting

Nursing students who were studying in different higher educational settings/institutions in the Southern Province were taken as the study settings: Faculty of Allied Health Sciences (FAHS), University of Ruhuna (UoR), and three (03) schools of nursing in Galle, Matara, and Hambantota. Final-year nursing undergraduates of the UoR and other nursing students at schools of nursing were selected using stratified sampling methods. Students who were able to read and understand Sinhala and the English language were the inclusion criteria. Students who were studying in academic years other than a final year were unable to give informed written consent with sufficient physical and psychological stability and were unable to read and understand Sinhala or English language exclusion criteria.

### Participants and sampling

The sample size of the study was calculated based on the formula (n = Z^2^ P (1-P) /d^2^ [26] [n = size of the sample, Z = 1.96, P = anticipated population proportion, d = absolute precision required for the estimate to fall within given, percentage point of the proportion = 5%]. According to a study conducted in Nepal, 69% of medical students had adequate knowledge of both first aid and snakebite. Therefore, 69% was taken as the proportion [27] The necessary minimum sample size was 384.16. After adding 10% for dropouts, the final study sample was 422 nursing students. All final year nursing undergraduates who studied in the Department of Nursing (n^1^ = 50), Faculty of Allied Health Sciences, University of Ruhuna and three schools of nursing—Galle (n^2^ = 125), Matara (n^3^ = 125), and Hambantota (n^4^ = 125) were included in one register, including their e-mail address after contacting relevant personnel and obtaining their permission.

### Study instruments

Data collection for the study was done using a pre-tested and self-administered questionnaire including three sections: socio-demographic characteristics, identification of venomous snakes, and first aid methods of snakebites. Items/questions in the questionnaire were prepared using previous literature and according to the guidelines of the WHO [20,23,24,27–31].

#### Identification of venomous snakes

**Fig 1:**
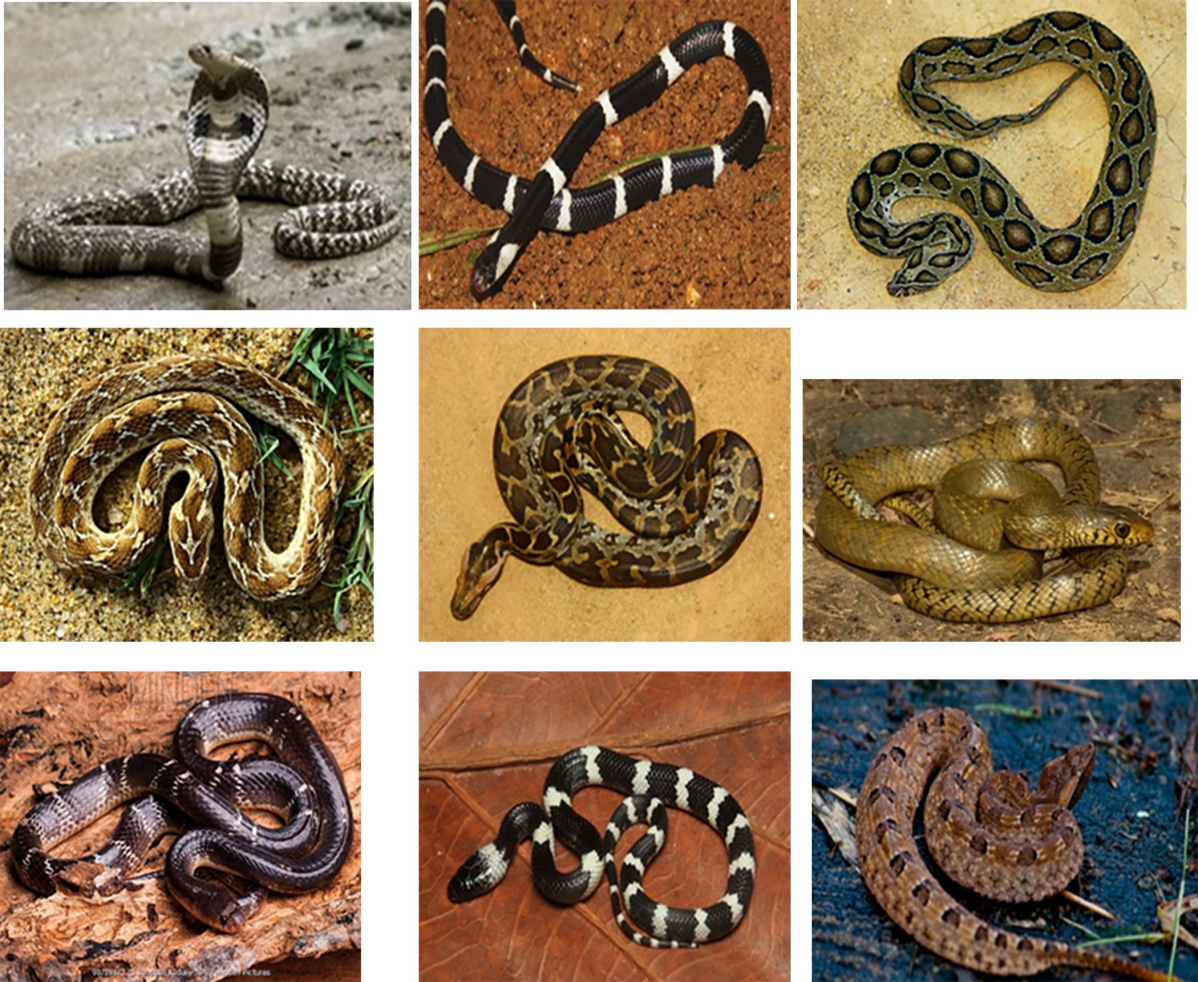
Images for the identification of snakes. Images of snakes-Image 1, Image 2, Image 3, Image 4, Image 5, Image 6, Image 7, Image 8, and Image 9.

Images for the identification of snakes were taken from the Google website (https://snakesidentification.org/) [3,32]. The questionnaire included 34 questions regarding identification associated with snakebites in addition to the images of different snakes (Table 1, Fig 1). Further, questions were assessed using a scoring method (score 1 = correct answer and 0 = incorrect or uncertain answer) prepared by the authors. The total score of overall knowledge of identification ranged from 0 to 34. Respondents who had scored levels 26 to 34 were considered as “High” knowledge, scores 13 to 25 were considered “Moderate” knowledge and scores 0 to 12 were considered “Low” knowledge.

**Table 1:** List of snakes and their venomous status.

No	Snake	Name of snakes (Sinhala name/s)	Scientific name	Envenoming status
1	Image 1	Cobra (Naya/Nagaya)	*Naja naja*	Highly venomous
2	Image 2	Ceylon krait (Dunu karawala/ Polon karawala/ Mudu karawala)	*Bungarus ceylonicus*	Highly venomous
3	Image 3	Russell’s viper (Thith polonga/ Dhara polonga)	*Daboia russelii*	Highly venomous
4	Image 4	Saw scaled viper (Weli polonga)	*Echis carinatus*	Highly venomous
5	Image 5	Python (Pimbura)	*Pythonidae*	Non-venomous
6	Image 6	Rat snake (Geradiya)	*Pantherophis obsoletus*	Non-venomous
7	Image 7	Common krait (Thel karawala/ Magamaruwa/ Habaralaya/ Mavilla)	*Bungarus caeruleus*	Highly venomous
8	Image 8	Wolf snake (Radanakaya)	*Lycodon aulicus*	Non-venomous
9	Image 9	Hump-nosed viper (Kunakatuwa/ Polonthelissa)	*Hypnale* spp.	Highly venomous

Images of snakes included in S1 Table.

#### First aid methods

The questionnaire included 16 questions regarding first aid methods associated with snakebites. Further, questions were finalized using a self-prepared scoring system, with a score of 1 for a correct response and 0 for an incorrect/uncertain response. The total correct responses were calculated to show the overall score of knowledge (score range 0 to 16). Respondents who had a score level of 12 to 16 were considered as “High” knowledge, scored 6 to 11 were considered “Moderate” knowledge, and those who scored 0 to 5 were considered “Low” knowledge. In addition, participants were asked whether they had received any training or not.

### Data collection

Data collection was performed after obtaining permission from the heads of the departments and principals of three schools of nursing. Recruitment and completion of the data collection was done during September 2022- November 2022. Socio-demographic and other relevant information were collected from nursing students using a Google Form questionnaire. Instruction was given via Google Forms, and all completed questionnaires were collected by e-mail. The medium of the questionnaire was English and Sinhala, and participants had a choice of language selection. The e-mail addresses of nursing students were received after obtaining permission from the relevant authorities. Thereafter, the link to the Google Form was distributed among them via e-mails. The Google Form consisted of two sections: an information sheet an informed written consent form, and a questionnaire. The information sheet provided a clear explanation of the study.

### Statistical analysis

Statistical packages of social sciences (SPSS) and Microsoft Excel were used for the data analysis and explained using pie charts, tables, histograms, and bar charts. The distribution of data was used to find the median, mean, frequency, percentage, etc. All categorical variables of the study were analyzed using the Chi-square test. All results were regarded as statistically significant at p < 0.05.

### Ethical approval

The study protocol approval was obtained from the Ethics Review Committee, FAHS, UoR, Galle, Sri Lanka (Ref. no. 69.11.2021). Permission was obtained from the Dean/FAHS, UoR, Head/Department of Nursing, FAHS, UoR, and three schools of nursing- Galle, Matara, and Hambantota districts. The purpose, method, and voluntary participation of this study were explained to the participants. The participants were allowed to leave the study at any time within the study period and were informed that there were no disadvantages to withdrawing their consent. Informed written consent was obtained from each participant using Google Forms before participation. Participants were invited to join after getting informed consent using the Google Form. The rights of participants were respected, and the confidentiality of information was strictly maintained. Only the research team had access to the participants’ information, and to protect the personal information of the participants, all the collected data were encrypted and protected using a password, and an identification code was used to minimize exposure.

## Results

### Students’ characteristics

Most of the students (82.6%) were in the 24–26 age category and females. Most of the students (n = 375; 88.2%) were educated in schools of nursing and only 11.8% of students (n = 50) were educated in the University of Ruhuna (Table 2).

**Table 2:** Socio-demographic characteristics among nursing students (n = 425).

Characteristics	Categories	Frequency	Percentage
Age group (years)	21–23	44	10.4
	24–26	351	82.6
	27 above	30	7.1
Gender	Male	65	15.3
	Female	360	84.7
Ethnicity	Sinhala	410	96.5
	Tamil	6	1.4
	Muslim	8	1.9
	Other	1	0.2
Religion	Buddhist	402	94.6
	Hindu	6	1.4
	Islam	8	1.9
	Christian	9	2.1
Institute of Education	Department of Nursing/University of Ruhuna	50	11.8
	School of Nursing/Galle	125	29.4
	School of Nursing/Matara	125	29.4
	School of Nursing/Hambantota	125	29.4

### Identification of snakes and venomous status

When considering the identification of snakes using previous images, only 421(99.1%) and 331(77.9%) students were able to identify the Cobra (Naya) and Rat snake (Geradiya) respectively (Table 3). However, nursing students had no definite understanding of the rest of the snakes. Although many students 386(90.8%) knew that the Cobra (Naya) was highly venomous, the majority did not know that the Sri Lankan krait (Mudu karawala), Russell’s viper (Thith polonga), Saw scaled viper (Weli polonga), Common krait (Thel karawala) and Hump-nosed viper (Kunakatuwa) were highly venomous as well.

**Table 3:** Identification of snakes and their venomous status by the nursing students (n = 425).

Snakes (photo, name and venomous status)		Correct answer	Frequency	Percentage
Image 1	Name of the snake	Cobra (Naya)	421 386	99.1 90.8
	Venom status	Highly venomous		
Image 2	Name of the snake	Sri Lankan krait (Mudu karawala)	57 47	13.4 11.1
	Venom status	Highly venomous		
Image 3	Name of the snake	Russell’s viper (Thith polonga)	85 57	20 13.4
	Venom status	Highly venomous		
Image 4	Name of the snake	Saw scaled viper (Weli polonga)	80 61	18.8 14.4
	Venom status	Highly venomous		
Image 5	Name of the snake	Python (Pimbura)	179 38	42.1 8.9
	Venom status	Nonvenomous		
Image 6	Name of the snake	Rat snake (Geradiya)	331 237	77.9 55.8
	Venom status	Nonvenomous		
Image 7	Name of the snake	Common krait (Thel karawala)	31 63	7.3 14.3
	Venom status	Highly venomous		
Image 8	Name of the snake	Wolf snake (Radanakaya)	44 26	10.4 6.1
	Venom status	Nonvenomous		
Image 9	Name of the snake	Hump nosed viper (Kunakatuwa)	50 65	11.8 15.3
	Venom status	Highly venomous		

### Level of knowledge among students about the identification of snakebites

**Fig 2:**
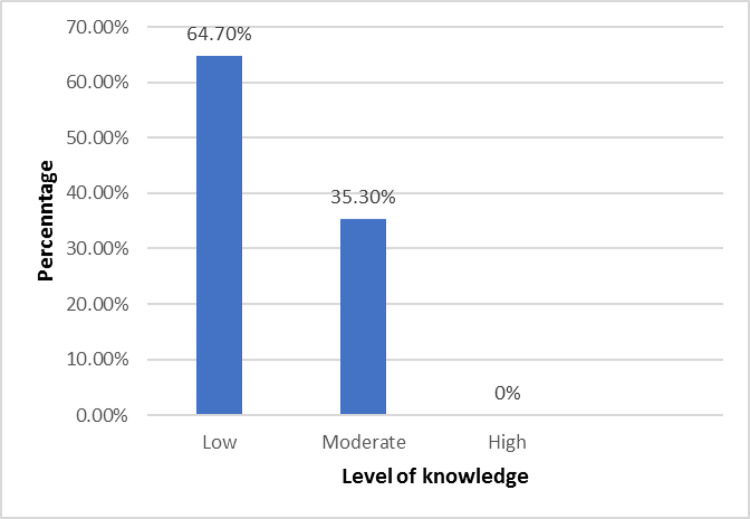
Level of knowledge on identification of snakebites among nursing students. Level of knowledge among nursing students about an identification of snakebites.

Most of the students (n = 275; 64.7%) had lower levels of knowledge on the identification of snakebites and there were no students with higher levels of knowledge on the identification of snakebites (Fig 2).

### Identification of clinical characteristics of venomous snakebites

Table 4 presents the general signs and symptoms after venomous snakebites which is important for the provision of first aid (percentage of correct responses). Most students knew that dizziness and vomiting, blurring of vision, difficulty in respiration, and severe muscle pain were common signs and symptoms of snakebites. On the other hand, a minority of them knew that unconsciousness, shock/collapse, and weakness of neck muscles as some signs and symptoms of snakebites.

**Table 4:** Identification of signs and symptoms associated with snake bites (n = 425).

Signs & Symptoms	Frequency	Percentage
Bleeding from gum and vomiting	181	42.6
Blurring of vision	262	61.6
Convulsion	109	25.6
Dark-colored urine	117	27.5
Difficulty in breathing	233	54.8
Difficulty in swallowing	110	25.9
Dizziness and vomiting	268	63.1
Heaviness of eyelids	170	40
Nasal regurgitation	165	24.7
Persistent bleeding from the bite site	172	40.5
Scanty or no urine output	197	46.4
Severe muscle pain	227	53.4
Shock/collapse	72	16.9
Swelling with pain and blistering	182	31.1
Unconsciousness	58	13.6
Weakness of neck muscle	96	22.6

### Knowledge level about first-aid methods after snakebites

**Fig 3:**
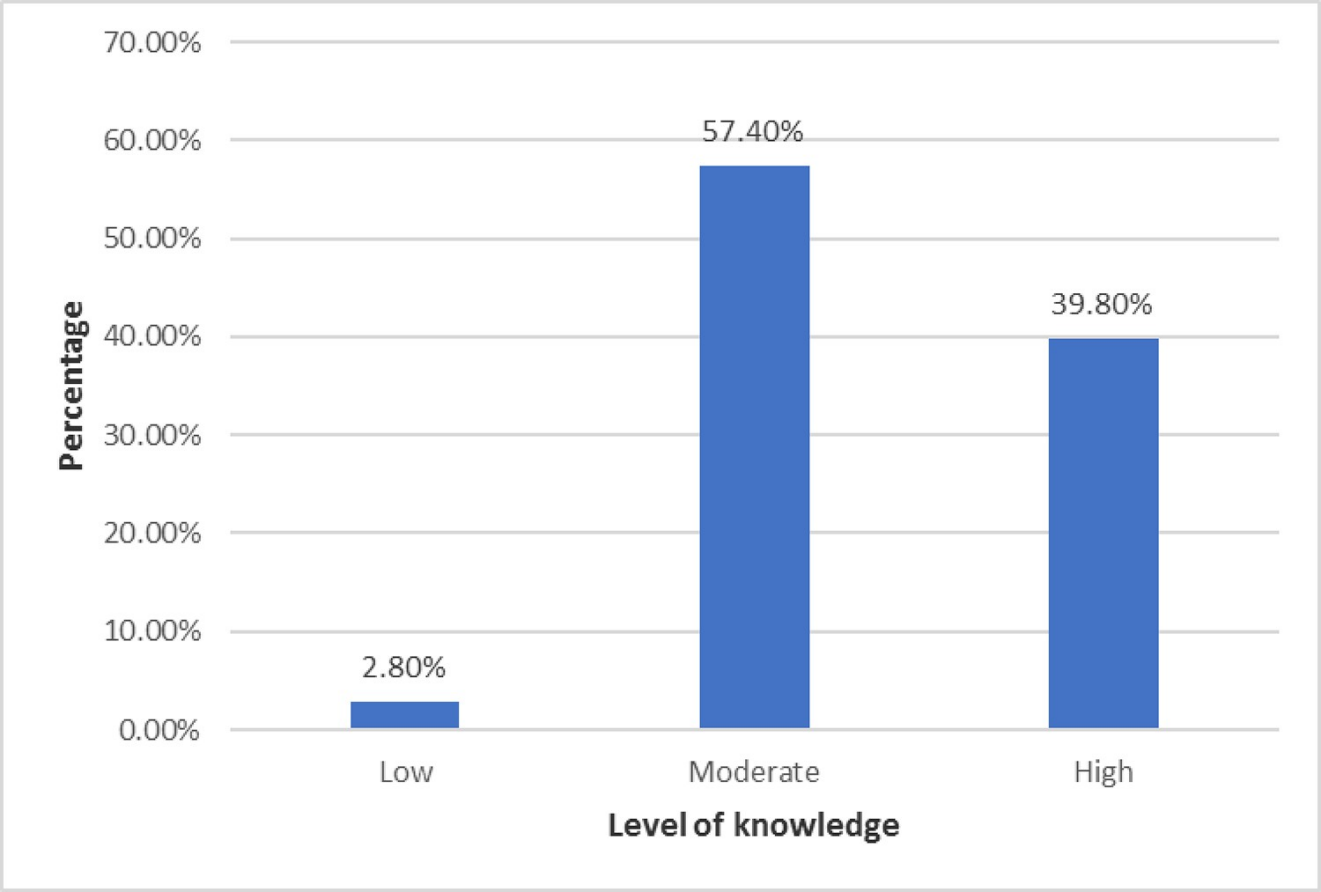
Level of knowledge on first aid method of snakebites among nursing students. Level of knowledge among nursing students about first aid methods of snakebites.

More than half of the students (n = 244; 57.4%) had moderate knowledge about first aid methods associated with snakebites and 169 participants (39.8%) had high knowledge regarding the first aid methods (Fig 3).

The first aid methods associated with snakebites are presented in Table 5 (percentages of the correct responses).

**Table 5:** Correct responses of nursing students on the knowledge of first aid methods associated with snakebites (n = 425).

Questions	Frequency (percentage)
Is telling the victim to stay calm beneficial?	378 (88.9)
Should snakebite patients be transported to the hospital soon after the bite?	406 (95.5)
Can antivenom therapy cure envenomation?	337 (79.3)
Should the wound of the bite site be rinsed (not scrubbed) with water as soon as possible?	374 (88)
Are all snake bites associated with envenomation?	320 (75.3)
Should pressure immobilization bandages be applied around the bite site?	117 (27.5)
Should healthy volunteers suck the venom out of the wound?	360 (84.7)
Should massage of the bite wound be done?	354 (83.3)
Should local incisions or pricks/punctures be made over the bite site?	377 (88.7)
Is electric current at the site of helpful bite?	346 (81.4)
Should the site of the bite be raised above the level of the patient’s heart?	191 (44.9)
Is the application of alcohol at the site of the bite beneficial?	299 (70.4)
Should tight bands(tourniquets) be applied around the limb proximal to the bite site?	222 (52.2)
Topical instillation or application of herbs beneficial?	136 (32)
Is the application of an ice pack at the site of the bite beneficial?	170 (40)
Analgesics should not be performed for first aid	151 (35.5)

Most students knew that informing the patient to stay calm is beneficial (n = 378; 88.9%) and were aware that those patients should be sent to the hospital as early as possible after the snakebite (n = 406; 95.5%), whereas only 117 (27.5%) knew that pressure immobilization bandages should not be applied around the bite site.

Further, there was a significant impact on students’ knowledge of first aid (Table 6). Among all participants, only 42% of nursing students had obtained several slots of training in working with snakebites.

**Table 6:** Impact of knowledge of students on first aid methods.

Variables		Knowledge of first aid method			Chi value	*p-value*
		Low	Moderate	High		
How students rate their knowledge	Good	2(0.5%)	24(5.6%)	30(7.1%)	15.083	*0*.*005*
	Average	6(1.4%)	180(42.4%)	126(29.6%)		
	Poor	4(0.9%)	40(9.4%)	13(3.1%)		

## Discussion

This survey was done to evaluate the knowledge in identifying venomous snakes and first aid methods for snakebites among nursing students in the Southern Province of Sri Lanka which would be very important.

Among the 425 nursing students, most of the sample (82.6%) represented the 24–26 age group and the majority was female (84.7%). In a study of the Lao People’s Democratic Republic, the mean age (±SD) of the sample was 37.0 (±10.9), and their age ranged from 22 to 56 years [23]. Sixty-two (52.1%) students were in the 20-35-year age group and the majority was female in the current study (73%). More than half of that study were physicians (n = 62, 52.1%), whereas 57 (47.9%) were nurses. When considering experiences, 62 (52.1%) participants had less than 10 years of experience, 18 (15.1%) participants had 10–20 years of practice, and 39 (32.8%) had more than 20 years, whereas nursing students of the current study were in their training period and had no such experiences. In the Lao study, most of the members (79, 66.4%), did not receive any training about the management of snakebites, whereas 40 participants (33.6%) reported that they did not receive training [23]. Only 42% of nursing students in the current study obtained some training in the management of snakebites.

A study conducted in China decided to examine the knowledge about snakes and snakebites among Chinese military members which were different from healthcare providers [20]. All were male military personnel (age ranges from ≤ 20–40) who engaged in a special troop in southeast China. Investigators intended to increase the medical education of military personnel and to reduce morbidity and mortality due to snakebites. That study exhibited that military members usually obtained information on snakebites through military medical education (89.7%), books/ magazines, newspapers (13.5%), television (8.5%), family-friends (8.4%), and the Internet (7.5%), to retrieve data [20]. When compared with the current findings, age, experiences, and training programs about snakebites would more impact the knowledge of the participants which could not be observed in this study as all were among current nursing students [20,23].

In this study, more than 75% of nursing students were able to identify Cobra (Naya) and Rat snake (Gerandiya) commonly seen in many areas in Sri Lanka. Most of the nursing students couldn’t correctly identify some of the snakes such as the Sri Lankan Krait, Common Krait, and Wolf Snake. Students had lower knowledge of identifying Russell’s Viper, Saw Scaled Viper, and Python. It was clear that students were not able to recognize images of snakes correctly because they had low knowledge of the characteristics of snakes. A study in Palestine revealed that the Palestine viper (Vipera palaestinae), a member of the viper family, is the most popular venomous snake in Palestine, accountable for most envenomation in humans (e.g., agricultural workers and children) and domestic animals [33].

In the Lao study, 72.3% (n = 86) had inadequate knowledge regarding snake identification. However, it was at a satisfactory level among the current nursing students. As mentioned in the previous study, adequate knowledge of snake identification was higher among different groups such as females and physicians, who were aged 36–50 years and had 10–20 years of experience, and who had received training at urban hospitals than the rural hospitals. The study sample’s knowledge of snake identification was significantly improved after getting training compared to those members who did not get any training [20,23]. Only 42% of nursing students had obtained training about snakebites in the current study.

Chen et al. [20] reported that the general level of knowledge regarding snakebites among field groups/military personnel in this study was not adequate. Most of the nursing students in the current study had low levels of knowledge of identification associated with snakebites.

Regarding some of the signs and symptoms of snakebites, many nursing students reported that dizziness and vomiting, blurring of vision, difficulty in respiration, and severe muscle pain were more common signs and symptoms of snakebites. On the other hand, a smaller number of students reported that unconsciousness, shock/collapse, and weakness of neck muscles could be some features of snakebites. As in the Lao study, more than 60% of participants had reported some signs and symptoms such as coagulation disorders, muscle paralysis with respiratory failure, and ptosis. Most participants properly responded that doing incision and suction, massaging the bite site, placing tourniquets, and administering the intramuscular injection of analgesics, were not advisable when handling patients with snakebites [23].

In a study in Bangladesh, knowledge of signs and symptoms of venomous snakebite among medical/health service providers was investigated [24]. It was reported that the doctors were able to mention some of the signs and symptoms (72.0% - 94.0%) and around 50% - 90% of the signs and symptoms of snake bite revealed by the nurses and some of the practitioners showed signs and symptoms ranging from 54.0% - 89.0%. Further, they emphasized that qualified doctors had more knowledge about some features of snakebite victims than those of the knowledge of nurses and other practitioners. However, results indicated that healthcare personnel were not aware of all attributes. Consequently, refresher programs and practical classes/aspects may be communicated to medical experts to understand the features of venomous and non-venomous snakebites simply and successfully [24].

First aid intends to delay the general absorption of venom, minimize dangerous and stressful early features of envenoming, preserve life early, avoid complications, and ensure victims obtain medical care. First aid measures have been recommended for each hurricane season/hurricane-stricken areas that worked in the emergency-preparedness deployment teams in North America [9].

The training in inappropriate first aid was recognized as one of the main challenges in ensuring proper care [20]. The incidence and deaths owing to snakebites could be controlled and minimized with sufficient medical intervention in many developing countries [34].

Their participants had a lower level of knowledge about first aid methods. A total of 73.8% of the studied military workers were informed to not move the whole of the victim’s body, precisely the bitten limb, which is a suitable practice for declining venom absorption [3]. However, 93.5% of participants emphasized that applying a tourniquet was ideal [20]. This percentage was higher in the previous studies conducted in Sri Lanka [6,35]. As mentioned by Silva et al. [6], farmers in the dry zone in Sri Lanka also had awareness of the most recommended first aid measures such as immobilizing the bitten part (89.5%); the bitten part should not be excised (79.7%); and alcohol should not be given (89%), etc. More than half of the nursing students in the current study had moderate knowledge about first aid methods associated with snakebites while 39.8% of participants had high knowledge regarding the first aid methods. While many participants knew that informing victims to stay calm was important and that snakebite patients should be taken to the hospital very quickly after the snakebite, only (27.5%) knew that pressure bandages should not be applied to such areas. According to WHO standards, vigorous washing should be prevented as this may boost the absorption of the venom and local bleeding.

Alcoba et al. [28], recommended having first aid training for healthcare professionals and traditional healers in Cameroon whereas Yousefi et al. [36] recommended expanding general awareness of snakebites among people/victims and increasing education of general/local people in highly prevalent areas would be more important. To prevent serious health consequences of snakebites, healthcare providers should be educated/trained on snakebite management [3]. Further, Hattimy et al. [25] reported the identification of high-risk regions consisting of snakebites, planning more awareness programs, improving the skills of medical staff on first aid and necessary care of patients with snakebites to decrease the morbidity and mortality related to snakebites and envenomation in Morocco. Courses or programs for healthcare providers should be conducted periodically as mentioned by Tochie et al. [16] which exhibited the different practices worldwide.

## Conclusions

The overall knowledge of the identification of venomous snakes among the nursing students was inadequate. This was the first study demonstrated in the Southern Province of Sri Lanka to assess nursing students’ knowledge regarding snakebites. Knowledge of first aid methods was at a moderate level. Approaches may need to enhance knowledge in identifying venomous snakes and first-aid methods of snakebites amongst nursing students in both educational settings. Therefore, conducting training courses on identification and first aid is recommended. To get the overall picture, another research should be done as a multi-center study incorporating all schools of nursing and universities in Sri Lanka. Future studies are suggested to evaluate other factors related to identification knowledge and first aid practices.

## Supporting information

S1 Table List of snakes and their venomous status (with images). (DOCX)

S1 File (PDF)

S2 File Questionnaire knowledge on first aid methods. (PDF)

S3 File (ZIP)
