# Supplementary material for: Knowledge in identifying venomous snakes and first aid methods of snakebites among nursing students: A cross-sectional study
Source: PLoS One. 2024 Apr 4;19(4):e0299814. doi: 10.1371/journal.pone.0299814 (PMC10994310; doi:10.1371/journal.pone.0299814)
Supplement: S1 Table — (DOCX) [file pone.0299814.s001.docx]

**S1 Table. List of snakes and their venomous status (with images)**

| **No** | **Snake** | **Name of snakes**  **(Sinhala name/s)** | **Scientific name** | **Envenoming status** |
| --- | --- | --- | --- | --- |
| 1 | 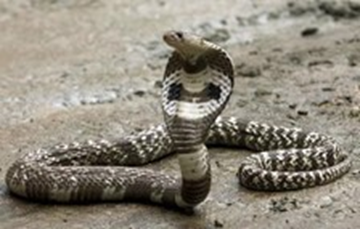 | Cobra (Naya/Nagaya) | *Naja naja* | Highly venomous |
| 2 | 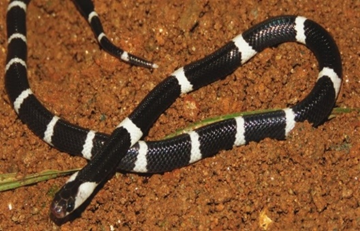 | Ceylon krait (Dunu karawala/ Polon karawala/ Mudu karawala) | *Bungarus ceylonicus* | Highly venomous |
| 3 | 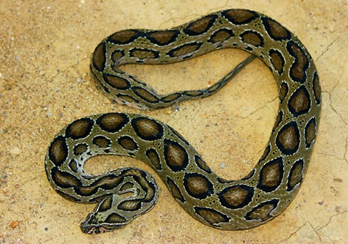 | Russell’s viper  (Thith polonga/ Dhara polonga) | *Daboia russelii* | Highly venomous |
| 4 | 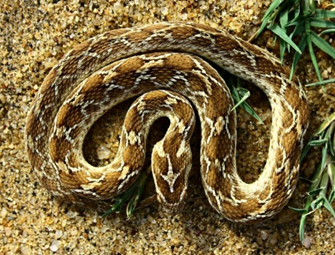 | Saw scaled viper  (Weli polonga) | *Echis carinatus* | Highly venomous |
| 5 | 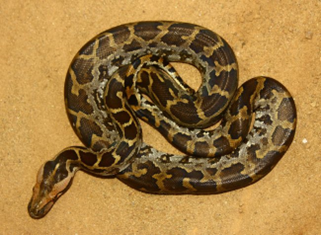 | Python  (Pimbura) | *Pythonidae* | Non-venomous |
| 6 | 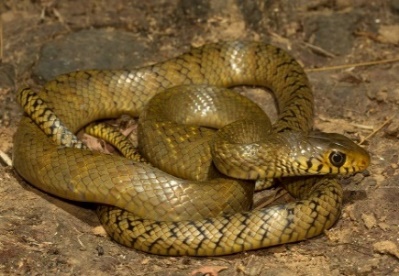­­­­ | Rat snake (Geradiya) | *Pantherophis obsoletus* | Non-venomous |
| 7 | 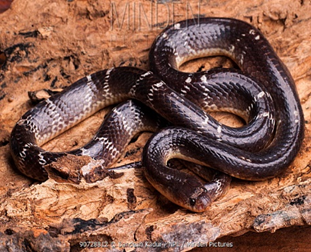 | Common krait (Thel karawala/ Magamaruwa/ Habaralaya/  Mavilla) | *Bungarus caeruleus* | Highly venomous |
| 8 | 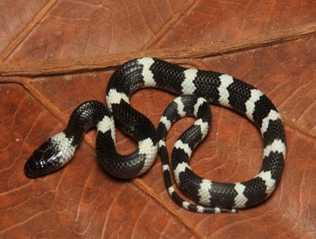 | Wolf snake  (Radanakaya) | *Lycodon aulicus* | Non-venomous |
| 9 | 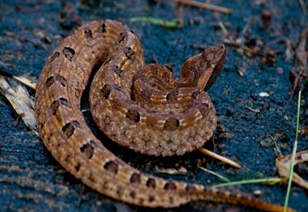 | Hump-nosed viper  (Kunakatuwa/  Polonthelissa) | *Hypnale* spp. | Highly venomous |
