# Supplementary material for: Knowledge in identifying venomous snakes and first aid methods of snakebites among nursing students: A cross-sectional study
Source: PLoS One. 2024 Apr 4;19(4):e0299814. doi: 10.1371/journal.pone.0299814 (PMC10994310; doi:10.1371/journal.pone.0299814)
Supplement: S2 File — (PDF) [file pone.0299814.s003.pdf]

Knowledge on first aid methods, diagnosis, and management of snakebites among nursing students in Southern Sri Lanka: a cross-sectional study

Instructions

Please fill out this questionnaire on your knowledge. Please refrain from seeking answers from other sources (Internet/books). You are free to fill in either the English or Sinhala version of the questionnaire.

Date: [ ] Serial No: [ ]

Section 01. Socio-demographic characteristics among nursing students

Socio-demographic details of Nursing Students.

Mark (✓) for the best response to relevant questions.

Write a suitable answer in the space given in other questions.

1. Age (Years) [ ]

2. Gender
   - Male [ ]
   - Female [ ]

3. Nationality
   - Sinhala [ ]
   - Tamil [ ]
   - Muslim [ ]
   - Other [ ]

4. Religion

- Buddhist
- Christian
- Hindu
- Islamic
- Other

5. Institute of Education

- Department of Nursing, Faculty of Allied Health Sciences, University of Ruhuna
- School of Nursing-Galle
- School of Nursing- Matara
- School of Nursing --Hambantota

Section 02. Self-assessment on Knowledge

Mark (✓) for the best response to relevant questions.

01.“How would you rate your knowledge about snakebite?”

- Good
- Average
- Poor

02.“How would you rate your demands for knowledge about snakebite?”

- High
- Moderate
- Low

03. Have you ever experienced snakebite?

- Yes ☐
- No ☐

04. Has a member of your family ever experienced snakebite?

- Yes ☐
- No ☐

05. Did you receive training about dealing/caring with snakebite patients?

- Yes ☐
- No ☐

06. Do you think that there is a need for training on snakebite management?

- Yes ☐
- No ☐

07. Do you think that there is an adequate facility in hospitals for snakebite management?

- Yes ☐
- No ☐

08. Where did you obtain the knowledge of snakebite?

- Medical/current education ☐
- Television ☐
- Books/magazines/newspapers ☐
- Families/friends ☐
- Internet ☐

09. What would your first reaction be if you faced snakebite?

Too nervous about doing anything	
Call for a surgeon or medical colleague	
Take simple interventions immediately	

Section 03. First Aid of Snakebites

Mark (✓) for the best response to relevant questions.

No.	Questions	Yes	No	Do not know
1.	Is telling the victim to stay calm beneficial?			
2.	Should snakebite patients be transported to the hospital soon after the bite?			
3.	Can antivenom therapy cure envenomation?			
4.	Should the wound of the bite site be rinsed (not scrubbed) with water as soon as possible?			
5.	Are all snake bites associated with envenomation?			
6.	Should pressure immobilization bandages be applied around the bite site?			
7.	Should healthy volunteers suck the venom out of the wound?			
8.	Should massage of the bite wound be done?			
9.	Should local incisions or pricks/punctures be made over the bite site?			
10.	Is electric current at the site of helpful bite?			
11.	Should the site of the bite be raised above the level of the patient’s heart?			
12.	Is the application of alcohol at the site of the bite beneficial?			
13.	Should tight bands(tourniquets) be applied around the limb proximal to the bite site?			
14.	Topical instillation or application of herbs beneficial?			
15.	Is the application of an ice pack at the site of the bite beneficial?			
16.	Analgesics should not be performed for first aid			

Section 04. Diagnosis of Snakebites

The following questions are about the diagnosis of snake bites

Identify the snakes and their venom status given below.

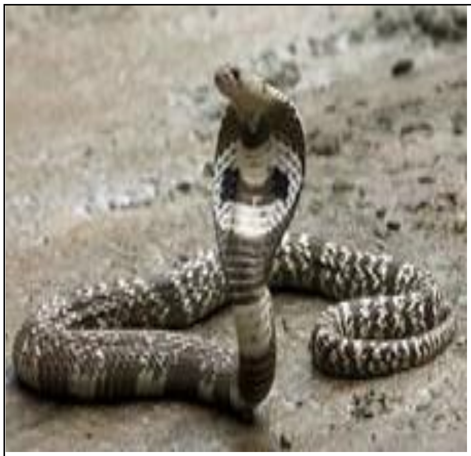	Cobra (Naya)<br>Common krait (Thel Karawala)<br>Sri Lankan krait (Mudu Karawala)<br>Russell's viper (Thith Polanga)<br>Saw scaled viper (weli Polanga)<br>Hump nosed viper (Kunakatuwa)<br>Python (Pimbura)<br>Rat snake (Geradiya)<br>Wolf snake (Radanakaya)<br>Do not know	Highly venomous<br>Mildly venomous<br>Nonvenomous<br>Do not know
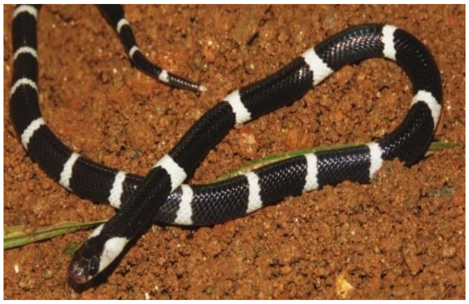	Cobra (Naya)<br>Common krait (Thel Karawala)<br>Sri Lankan krait (Mudu Karawala)<br>Russell's viper (Thith Polanga)<br>Saw scaled viper (weli Polanga)<br>Hump nosed viper (Kunakatuwa)<br>Python (Pimbura)<br>Rat snake (Geradiya)<br>Wolf snake (Radanakaya)<br>Do not know	Highly venomous<br>Mildly venomous<br>Nonvenomous<br>Do not know
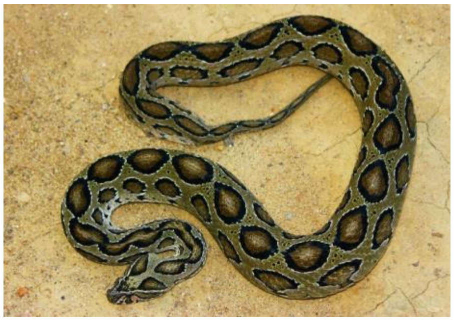	Cobra (Naya)<br>Common krait (Thel Karawala)<br>Sri Lankan krait (Mudu Karawala)<br>Russell's viper (Thith Polanga)<br>Saw scaled viper (weli Polanga)<br>Hump nosed viper (Kunakatuwa)<br>Python (Pimbura)<br>Rat snake (Geradiya)<br>Wolf snake (Radanakaya)<br>Do not know	Highly venomous<br>Mildly venomous<br>Nonvenomous<br>Do not know

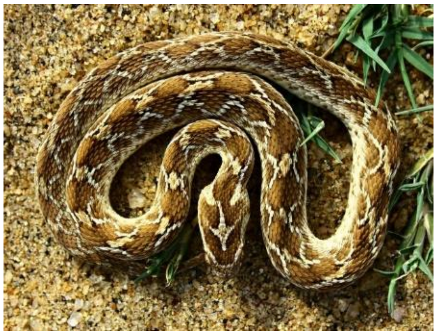	Cobra (Naya)		Highly venomous	
	Common krait (Thel Karawala)		Mildly venomous	
	Sri Lankan krait (Mudu Karawala)		Nonvenomous	
	Russell's viper (Thith Polanga)		Do not know	
	Saw scaled viper (weli Polanga)			
	Hump nosed viper (Kunakatuwa)			
	Python (Pimbura)			
	Rat snake (Geradiya)			
	Wolf snake (Radanakaya)			
	Do not know			
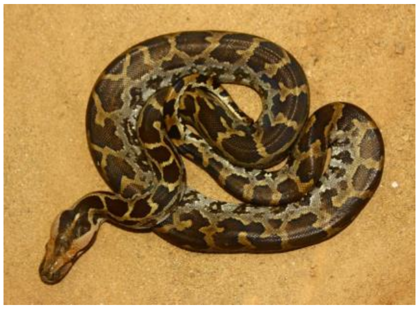	Cobra (Naya)		Highly venomous	
	Common krait (Thel Karawala)		Mildly venomous	
	Sri Lankan krait (Mudu Karawala)		Nonvenomous	
	Russell's viper (Thith Polanga)		Do not know	
	Saw scaled viper (weli Polanga)			
	Hump nosed viper (Kunakatuwa)			
	Python (Pimbura)			
	Rat snake (Geradiya)			
	Wolf snake (Radanakaya)			
	Do not know			
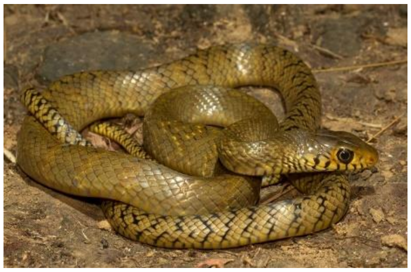	Cobra (Naya)		Highly venomous	
	Common krait (Thel Karawala)		Mildly venomous	
	Sri Lankan krait (Mudu Karawala)		Nonvenomous	
	Russell's viper (Thith Polanga)		Do not know	
	Saw scaled viper (weli Polanga)			
	Hump nosed viper (Kunakatuwa)			
	Python (Pimbura)			
	Rat snake (Geradiya)			
	Wolf snake (Radanakaya)			
	Do not know			

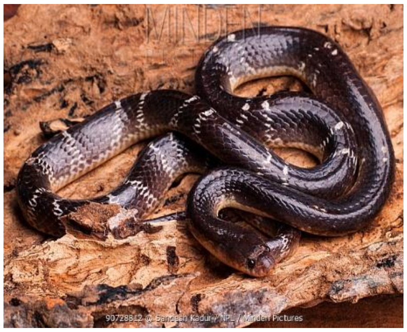

Cobra (Naya)	
Common krait (Thel Karawala)	
Sri Lankan krait (Mudu Karawala)	
Russell’s viper (Thith Polanga)	
Saw scaled viper (weli Polanga)	
Hump nosed viper (Kunakatuwa)	
Python (Pimbura)	
Rat snake (Geradiya)	
Wolf snake (Radanakaya)	
Do not know	

Highly venomous	
Mildly venomous	
Nonvenomous	
Do not know	

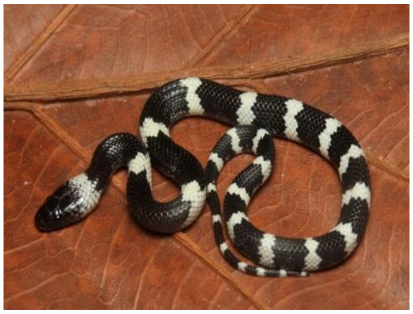

Cobra (Naya)	
Common krait (Thel Karawala)	
Sri Lankan krait (Mudu Karawala)	
Russell’s viper (Thith Polanga)	
Saw scaled viper (weli Polanga)	
Hump nosed viper (Kunakatuwa)	
Python (Pimbura)	
Rat snake (Geradiya)	
Wolf snake (Radanakaya)	
Do not know	

Highly venomous	
Mildly venomous	
Nonvenomous	
Do not know	

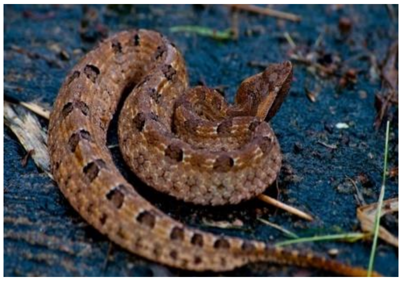

Cobra (Naya)	
Common krait (Thel Karawala)	
Sri Lankan krait (Mudu Karawala)	
Russell’s viper (Thith Polanga)	
Saw scaled viper (weli Polanga)	
Hump nosed viper (Kunakatuwa)	
Python (Pimbura)	
Rat snake (Geradiya)	
Wolf snake (Radanakaya)	
Do not know	

Highly venomous	
Mildly venomous	
Nonvenomous	
Do not know	

What are the signs and symptoms associated with snake bites?

No.	Signs & Symptoms	Yes	No	Do not know
1.	Bleeding from gum and vomiting			
2.	Blurring of vision			
3.	Convulsion			
4.	Dark-coloured urine			
5.	Difficulty in respiration			
6.	Difficulty in swallowing			
7.	Dizziness and vomiting			
8.	Heaviness of eyelids			
9.	Nasal regurgitation/voice93			
10.	Persistent bleeding from the bite site			
11.	Scanty or no urine output			
12.	Severe muscle pain			
13.	Shock/collapse			
14.	Swelling with pain and blistering			
15.	Unconsciousness			
16.	Weakness of neck muscle			

Part 04. Treatment of Snakebites

What are the investigations associated with snake bites?

No.	Investigations	Yes	No	Do not know
1.	20-minute whole blood clotting			
2.	Blood grouping and Rh typing			
3.	Blood urea (B.U)			
4.	Complete blood count (FBC)			
5.	Serum Creatinine (S. Cr)			
6.	Electrocardiogram (ECG)			
7.	Serum Electrolyte (S.E)			

8.	Immunodiagnostics			
9.	Serum Creatine Phosphokinase (CPK)			
10.	Urine routine examination(R/E)			

1. Capturing the offending snake for identification is not essential in treating the patient.

   Yes ☐

   No ☐

2. Snakebites can be successfully treated in Sri Lanka.

   Yes ☐

   No ☐

3. Antivenom is available only in some hospitals in Sri Lanka.

   Yes ☐

   No ☐

4. Which antivenom is used in Sri Lanka?

   Polyvalent ☐

   Monovalent ☐

5. Polyvalent antivenom is more useful when the offending snake has been identified.

   Yes ☐

   No ☐

6. Can antivenom be used for hump-nosed viper bites?

   Yes ☐

   No ☐

.

7. Polyvalent antivenom can be used for cobra, common krait, Russell's viper, and saw-scaled viper.

   Yes \ ☐

   No ☐

8. Where was the antivenom stored in your facility?

   Refrigerator ☐

   Others (shelf, room temperature) ☐

Part A

01. Do you think that there is a need to dilute antivenom before giving it?

   Yes ☐

   No ☐

02. Do you think that the required amount of antivenom varies with the severity of envenomation?

   Yes ☐

   No ☐

Part B

01. What is the best route to give antivenom injection?

   Intravenous (IV) ☐

   Intramuscular (IM) ☐

   Do not know ☐

02. How many vials must at least be available if the patient has been envenomated?

10 ☐
15 ☐
20 ☐
Do not know ☐

03. What do you think about the average rate of infusion for antivenom?

5 vials per 15 minutes ☐
10 vials per 15 minutes ☐
20 vials per 60 minutes ☐
Do not know ☐

04. The amount of antivenom needed for minor envenomated bites.

1-2 ☐
3–5 ☐
5–10 ☐
Do not know ☐

05. The amount of antivenom needed for moderate or severe envenomated bites.

5-10 ☐
10–15 ☐
15–20 ☐
Do not know ☐

Part C

01. What is the complication of antivenom therapy?

Anaphylaxis (urticaria, dyspnea, hypotension) ☐
Pyrogenic reaction (fever and chill) ☐
Diarrhea and vomiting ☐

Thank you for your valuable participation
