# Supplementary material for: Knowledge in identifying venomous snakes and first aid methods of snakebites among nursing students: A cross-sectional study
Source: PLoS One. 2024 Apr 4;19(4):e0299814. doi: 10.1371/journal.pone.0299814 (PMC10994310; doi:10.1371/journal.pone.0299814)
Supplement: S3 File — (ZIP) [file pone.0299814.s004.zip › Figure PDF/S5_Fig.pdf]

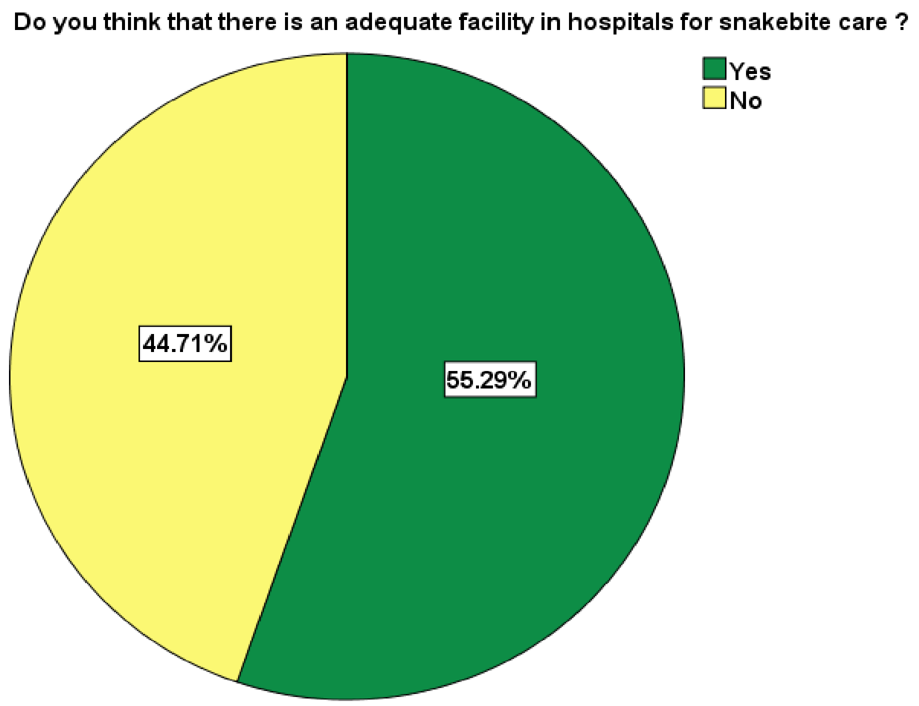

Fig 5. Adequacy of facilities in the hospital.
