# Supplementary figures and images for: Knowledge in identifying venomous snakes and first aid methods of snakebites among nursing students: A cross-sectional study
Source: PLoS One. 2024 Apr 4;19(4):e0299814. doi: 10.1371/journal.pone.0299814 (PMC10994310; doi:10.1371/journal.pone.0299814)

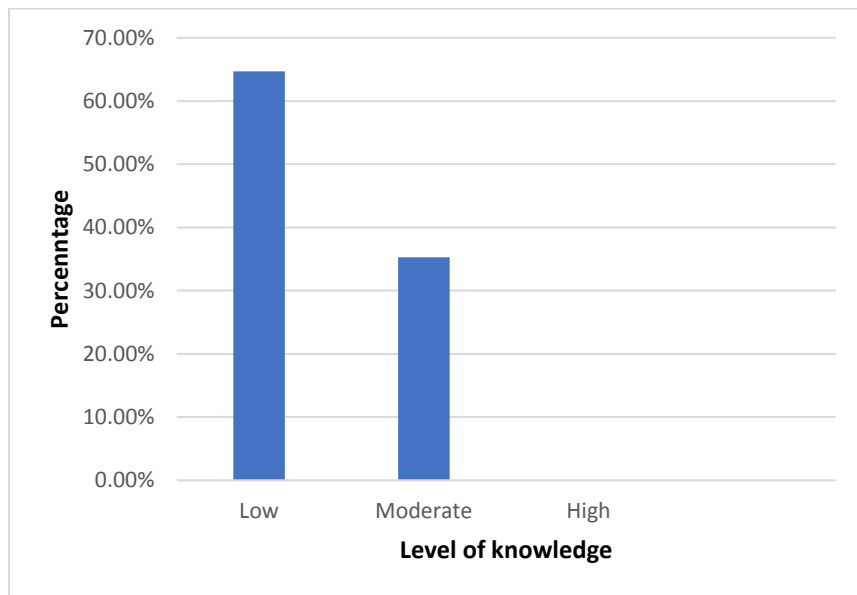

**Fig 1. Level of knowledge on identification of snakes and snakebites among nursing students.**

Supplement: S3 File — (ZIP) [file pone.0299814.s004.zip › Figure PDF/S1_Fig.pdf]

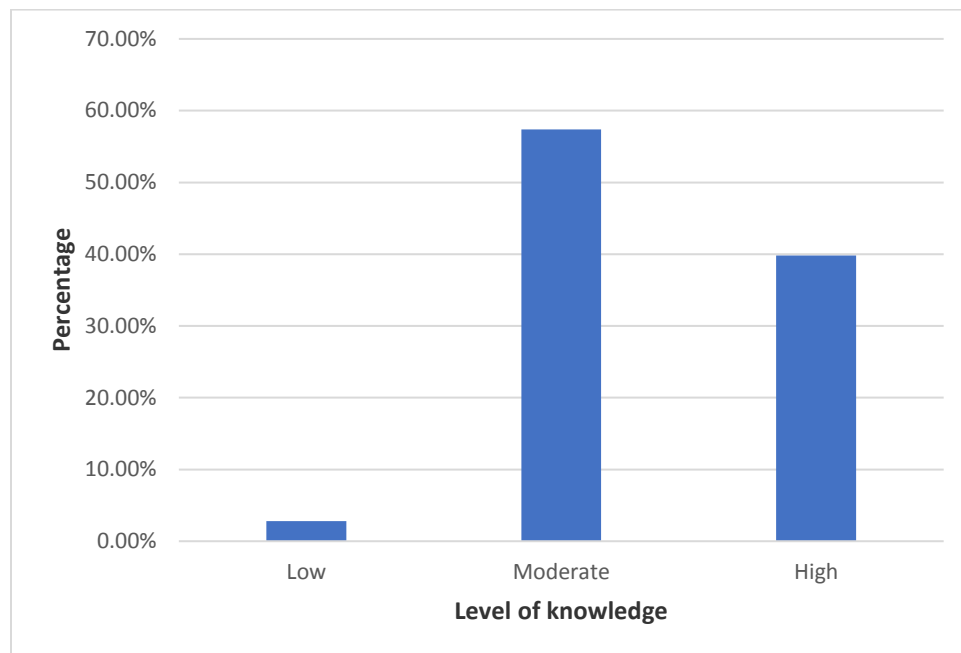

**Fig 2. Level of knowledge on first aid method of snakebites among nursing students.**

Supplement: S3 File — (ZIP) [file pone.0299814.s004.zip › Figure PDF/S2_Fig.pdf]

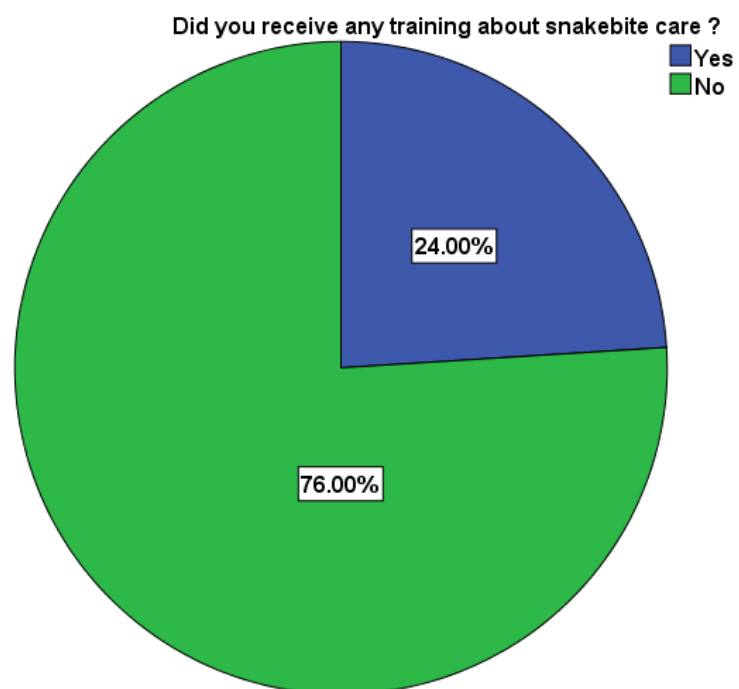

**Fig 3. Received training on snakebites.**

Supplement: S3 File — (ZIP) [file pone.0299814.s004.zip › Figure PDF/S3_Fig.pdf]

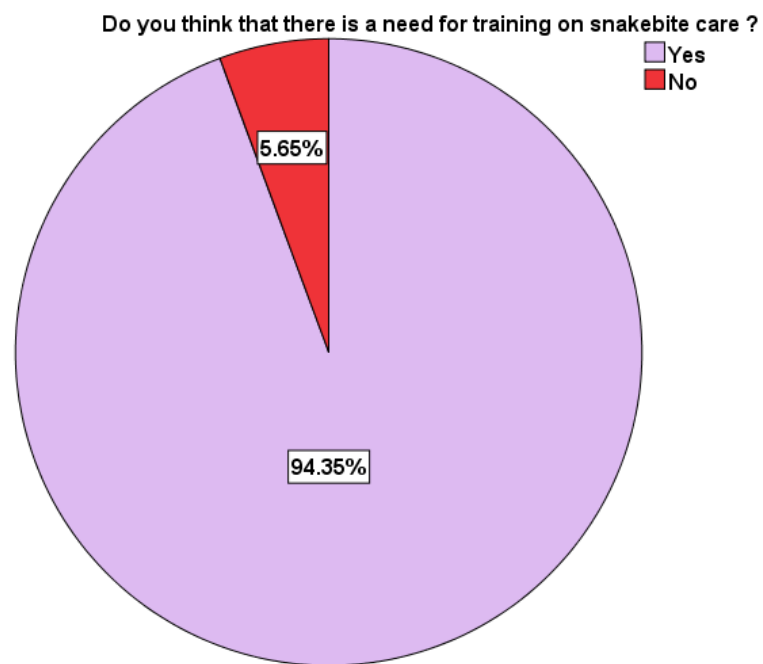

**Fig 4. Need for training.**

Supplement: S3 File — (ZIP) [file pone.0299814.s004.zip › Figure PDF/S4_Fig.pdf]

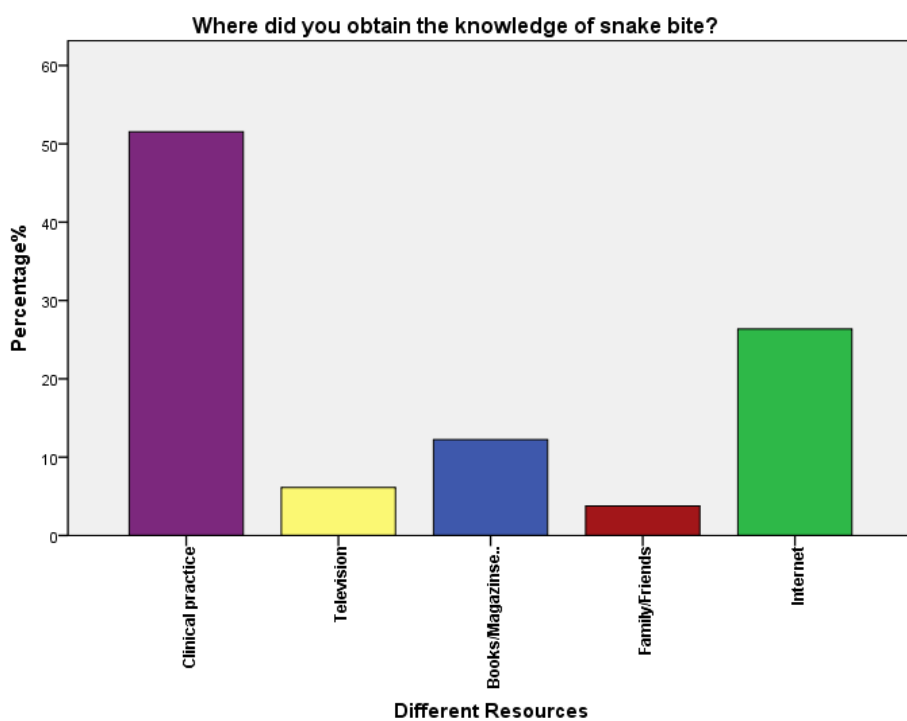

**Fig 6. Utilized resources.**

Supplement: S3 File — (ZIP) [file pone.0299814.s004.zip › Figure PDF/S6_Fig.pdf]
